# Co-crosslinking strategy for dual functionalization of small magnetic nanoparticles with redox probes and biological probes

**DOI:** 10.1007/s00604-024-06517-8

**Published:** 2024-07-05

**Authors:** Ye Chen, Feixiong Chen

**Affiliations:** 1 Huangyan District Center for Disease Control and Prevention, Taizhou, Zhejiang China; 2 Centre for BioNano Interactions, School of Chemistry and Chemical Biology, University College Dublin, Belfield, Dublin 4 Ireland; 3 Disease Networks Research Unit, Faculty of Biochemistry and Molecular Medicine, University of Oulu, 90014 Oulu, Finland

**Keywords:** Magnetic nanoparticles (MNPs), Surface functionalization, Bioconjugation, Electrochemical sensing, Point-of-care use

## Abstract

Surface functionalization strategy is becoming a crucial bridge from magnetic nanoparticles (MNPs) to their broad bio-application. To realize the multiple functions of MNPs such as magnetic manipulation, target capture, and signal amplification in their use of electrochemical biosensing, co-crosslinking strategy was proposed here to construct dual-functionalized MNPs by combining ultra-sensitive redox moieties and specific biological probes. In this work, MNPs with a TEM size of 10 nm were synthesized by co-precipitation for amination and PEGylation to maintain colloid stability once dispersed in high-ionic-strength buffer (such as phosphate-buffered saline). Then, MNPs@IgG were prepared via the bis(sulfosuccinimidyl) suberate (BS3) cross-linker to conjugate these IgG onto the MNP surface, with a binding efficiency of 73%. To construct dual-functionalized MNPs, these redox probes of ferrocene-NHS (Fc) were co-crosslinked onto the MNP surface, together with IgG, by using BS3. The developed MNPs@Redox@IgG were characterized by SDS‒PAGE to identify IgG binding and by square wave voltammetry (SWV) to validate the redox signal. Additionally, the anti-CD63 antibodies were selected for the development of MNPs@anti-CD63 for use in the bio-testing of exosome sample capture. Therefore, co-crosslinking strategy paved a way to develop dual-functionalized MNPs that can be an aid of their potential utilization in diagnostic assay or electrochemical methods.

**Graphical Abstract:**

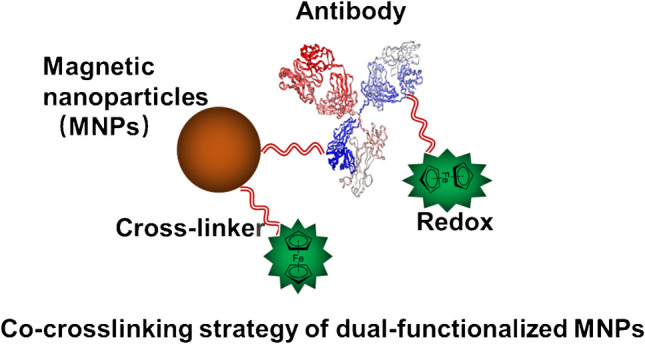

**Supplementary Information:**

The online version contains supplementary material available at 10.1007/s00604-024-06517-8.

## Introduction

Magnetic nanoparticles (MNPs) have been extensively employed in bio-applications by acting as magnetic carriers, signal amplifiers, or transducers to enhance their multi-roles for both separation and biosensing [1–3]. As MNPs have been comprehensively reviewed for electrochemical immunoassays and in developing electrochemical sensors [4–6], these MNPs offered remarkable advantages, such as an improved sensitivity, a low detection limit, a high signal-to-noise ratio, and a rapid analysis [7, 8], owing to their ease of size control, high surface-to-volume ratio, and unique magnetic manipulation. However, the bridge connecting MNPs to their electrochemical biosensor strongly depended on the progress of MNP’s surface functionalization strategies. On one side, typically, the attractive van der Waals and magnetic interparticle forces might cause agglomeration or precipitation [9], resulting in MNPs’ aggregation in biological media or phosphate-buffered saline (PBS). And these surface functionalization strategies can potentially address the dispersion stability of MNPs, particularly for these bare MNPs. On the other side, they can also provide a solution to combine these multi-functionalities of redox signal amplification or biological capture, for their use of biosensing [10–12]. Clearly, surface functionalization strategies played a significant role in the aid of wide MNP application.

For example, Budny et al*. *[13] have reported the conjugation of ferrocene (Fc) redox probes onto nanoparticle (NP) surfaces by covalent bonding strategy, whereas such Fc-functionalized NPs can be dispersed only in organic solvents, due to the highly hydrophobic surface of ferrocene. To obtain the stability of MNPs dispersion in aqueous solution, an alternative strategy was to coat the polyelectrolyte layers on the NP surface via layer-by-layer adsorption [14]. However, the insulating properties of these polyelectrolyte layers might reduce the electron transfer efficiency of these NPs for use in electrochemical biosensing, while the polyelectrolyte layers adsorbed at the NP surface might be easily destabilized and be released from the NP surface in high-strength ionic buffers of PBS, leading to NP aggregation. Additionally, although Feng et al*. *[15] and Li et al*. *[16] also worked on the conjugation of Fc probes and antibodies onto MNPs through chemical cross-linking, they were mainly concerned with electrochemical biosensing applications, and the detailed characterization and colloid stability of dual-functionalized MNPs were not well investigated in their study. Indeed, a robust surface functionalization strategy was still needed in developing dual-functionalized MNPs, particularly for evaluating MNP colloid stability via the characterization of these small MNPs from synthesis to each step of surface functionalization. Because such strategy enables to feature the NPs with magnetic, redox, and biological properties, playing their multiple roles in magnetic separation, target capture, and electrochemical amplification, with the potential to simplify the diagnostic process and enhance their diagnostic performance [17]. Meanwhile, it can aid the utilization of MNPs in the point-of-care (POC) assay, electroanalytical methods, or electrochemical biosensors [16, 17].

**Fig. 1:**
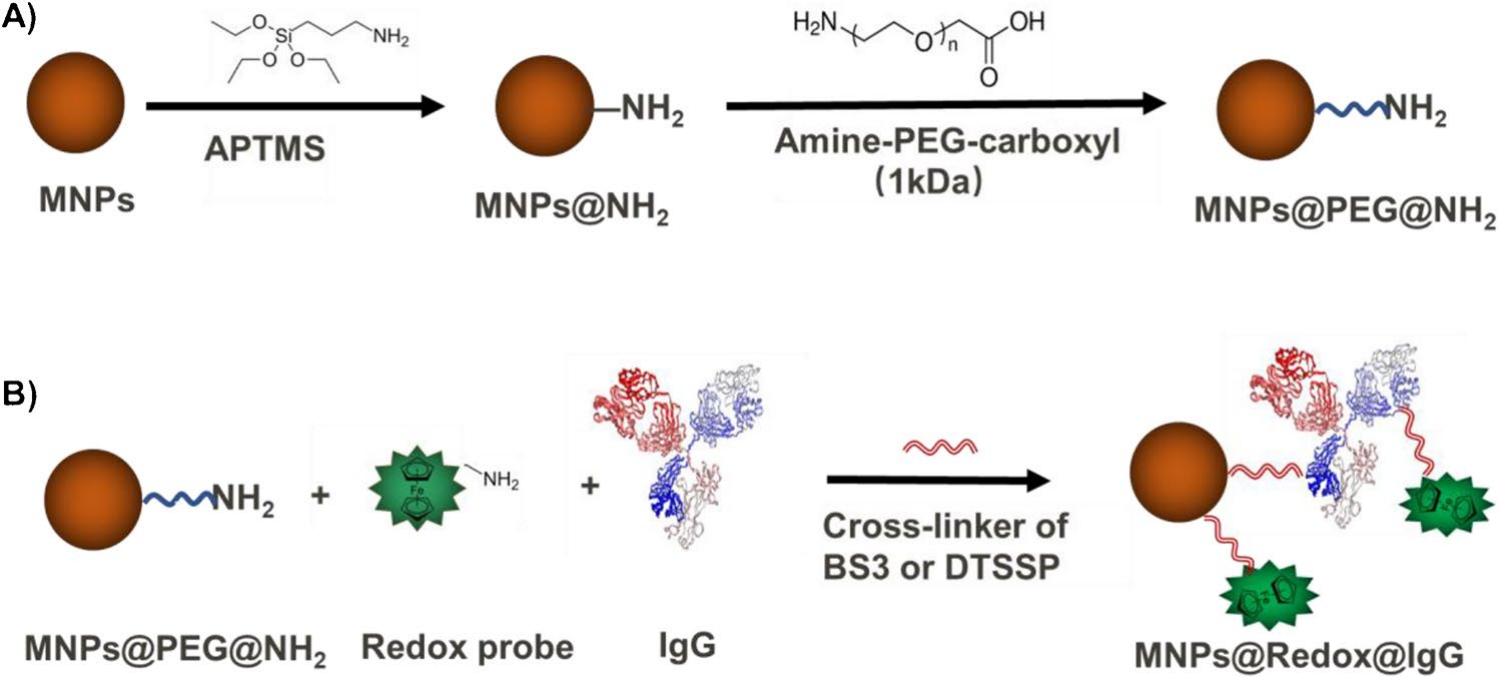
**A** Surface functionalization of magnetic nanoparticles (MNPs) with APTMS and amine-PEG-Carboxyl. **B** Co-crosslinking of IgG and redox probes onto the MNP surface

Herein, the co-crosslinking methods proposed here can be used to construct dual-functionalized MNPs capped with both redox and biological probes, where their surface functionalization process was characterized in detailed starting from the bare MNPs. Firstly, after MNP synthesis, as shown in Fig. 1A, the bare MNPs were firstly aminated by APTMS to graft the functional group of amine, while the obtained MNPs@NH_2_ were further PEGylated to MNP@PEG@NH_2_ by using amine-PEG-carboxyl to ensure that these MNPs maintained their colloidal stability in PBS buffer owing to their high density of surface charge. Secondly, to construct the dual-functionalized MNPs shown in Fig. 1B, the free amine-based redox probes and antibodies were co-crosslinked onto the MNP@PEG@NH_2_ surface through a homobifunctional cross-linker. On one side, human IgG with a molecular weight of 150 kDa was the second most abundant plasma protein in human blood, and it presented a typical antibody structure, comprising one heavy and one light chains linked by disulfide bridges [18, 19]. As the biomolecules probes, IgG was firstly selected to validate the co-crosslinking strategy. Then, the same strategy was extended to conjugate anti-CD63 instead of IgG, being ready for bio-testing. On the other side, the ferrocene group exhibited an excellent electroactivity and had been widely selected as an electrochemical signal probe [20–23]. Thus, we initially used the redox probe of ferrocene for this study, while this co-crosslinking strategy might further provide the possibility to construct other redox probes in MNP preparation, such as methylene blue or anthraquinone. Particularly, BS3 and DTSSP as the cross-linkers used in this strategy, which contained two terminal groups of NHS-ester, exhibited high reactivity toward free amine groups of biomolecules and can co-crosslink both the antibodies and the redox probes onto MNP@PEG@NH_2_. Lastly, via our co-crosslinking strategy, the prepared MNPs were further used for exosome extraction, validating their potential bio-application.

## Experiment and materials

Ferric chloride, ferrous chloride, (3-aminopropyl)trimethoxysilane (APTMS), N-hydroxysuccinimide (NHS), poly(amidoamine) dendrimer generation 0 (PAMAM G.0), immunoglobulin G (IgG), (3-aminopropyl)trimethoxysilane (APTMS), and N-(3-dimethylaminopropyl)-N′-ethylcarbodiimide hydrochloride (EDC) were purchased from Sigma-Aldrich. RIPA lysis buffer, bis(sulfosuccinimidyl) suberate (BS3), and 3,3′-dithiobis (sulfosuccinimidyl propionate) (DTSSP) were obtained from Thermo Fisher Scientific In. Amine-PEG-Carboxy (NH_2_-PEG-COOH,1.0 kDa) was obtained from Biochempeg (USA). N-Succinimidyl ferrocenecarboxylate (Fc-NHS) was purchased from Tokyo Chemical Industry UK Ltd. Anti-CD63 [TS63]—BSA and azide-free (ab59479) were purchased from Abcam. N-2-Hydroxyethylpiperazine-N′-2-ethanesulfonic acid (HEPES) and phosphate-buffered saline (PBS) were obtained from Sigma-Aldrich and were prepared in deionized (DI) water at a concentration of 10.0 mM at pH = 7.4. All chemicals were used as received without any further purification.

Spectral/Por®1 Dialysis membrane standard RC tubing with a MWCO of 6–8 kDa was obtained from Spectrum Laboratories, Inc. The LS columns were obtained from Miltenyi Biotec and used to concentrate the MNPs.

### Synthesis of small MNPs

As described in Cursi et al*. *[24], the small MNPs (iron (III) oxide, *γ*-Fe_2_O_3_) were synthesized by the co-precipitation method. Briefly, ferric chloride (0.030 mol) in 350.0 mL of Milli-Q DI water and ferrous chloride (0.015 mol) in 17.0 mL of hydrochloric acid (1.5 M) were vigorously mixed under mechanical stirring. Then, 30.0 mL of 35% ammonia solution was added to form the iron oxide nanoparticles. After washing three times in DI water, these iron oxides were further dispersed in 25.0 mL of nitric acid (2.0 M) for 15 min to partly oxidize the iron oxide surface. Finally, the NPs were mixed with 60.0 mL of iron nitrate (0.33 M) for 30 min to fully oxidize the iron(II, III) oxide to iron(III) oxide, which are these small MNPs used for the subsequent study.

### Amination of MNPs

A total of 5.0 mL of MNP solution at a concentration of 22.5 mg/mL was mixed with 10.0 mL of ethanol, and 0.36 mL of APTMS was added for incubation overnight. With the addition of 10.0 mL of glycerol, the mixture was allowed to react via amination at 95 °C and 45 mbar for 2 h under a rotary evaporator. After acetone washing, the MNPs@NH_2_ were collected in 5.0 mL of DI water. After removing water via the Schenk line, the MNPs@NH_2_ were stored in DMSO before use.

### PEGylation of MNPs@NH_2_

In 600.0 μL of DMSO, 0.0153 mmol of amine-PEG-carboxyl was activated by 0.045 mmol of EDC and 0.045 mmol of NHS and incubated for 45 min at 37 °C. Then, under the ratio control of 10.0 PEG per nm^2^ of MNP surface, 400.0 μL of MNP@NH_2_ (20.0 mg/mL in DMSO) was mixed with 600.0 μL of the abovementioned amine-PEG-carboxyl solution overnight at 37 °C and 550 rpm. After that, 1.0 mL of MNP@PEG@NH_2_ was directly diluted in 10.0 mL of DI water for membrane dialysis (6–8 kDa) overnight. With an LS magnetic column, the MNPs@PEG@NH_2_ were collected by a magnet and redispersed in DI water for use.

### Crosslinking of IgG onto MNPs@PEG@NH_2_

At a MNP/IgG ratio of 2.5, 300.0 μL amine-PEG-carboxyl (1.6 mg/mL) was mixed with 60.0 μL of IgG (0.63 mg/mL in PBS1X). Then, 60.0 μL of BS3 or 25.0 μL of DTSSP (10.0 mg/mL in water) was further added to start the crosslinking reaction, which was incubated for 30 min at 37 °C and 550 rpm. Finally, after the addition of 1.5 mL of PBS1X to quench the crosslinking process, the MNPs@IgG were washed with PBS three times via magnetic collection and redispersion in 200.0 μL of PBS1X for analysis and bio-testing.

### Conjugation of Fc-NHS to PAMAM dendrimer G.0

The redox probe Fc-NHS was dissolved in DMSO at a concentration of 10.0 mg/mL. For the preparation of G.0@Fc, different molecular ratios of Fc to G.0 (at 1, 2, 3, and 4) were prepared by adjusting the amount of Fc to PAMAM G.0, with the labels G.0@Fc-1, G.0@Fc-2, G.0@Fc-3, and G.0@Fc-4, respectively. For example of G.0@Fc-2 with a molecular ratio of Fc/G.0 at 2, 40.0 μL of PAMAM G.0 (20 wt. % in methanol) was mixed with 400.0 μL of Fc probes to prepare the probe G.0@Fc-2 for 2 h of incubation at 60 °C and 550 rpm.

### Redox labelling of MNPs by BS3 Co-crosslinking

Briefly, 300.0 μL of MNPs@PEG@NH_2_ (1.6 mg/mL), 5.0 μL of G.0@Fc-2 (10.0 mg/mL in DMSO), and 60.0 μL of IgG (0.63 mg/mL) were mixed together, and 60.0 μL of BS3 (10.0 mg/mL) was subsequently added again for 30 min of co-crosslinking incubation at 37 °C and 550 rpm (the ratio of Fc/MNPs surface is 1.0 per nm^2^). After washing, the MNPs@Redox@IgG were dispersed in 200.0 μL of PBS1X for characterization.

### Bio-testing of exosome samples

30.0 μL of MNPs@PEG@NH_2_ (1.6 mg/mL) was mixed with 6.0 μL of anti-CD63 (1.0 mg/mL) and 1.5 μL of G.0@Fc-4. Then, 8.0 μL of DTSSP (10.0 mg/mL) was mixed inside each tube for co-crosslinking at 37 °C and 550 rpm for 30 min. After the co-crosslinking process was quenched by the addition of 500.0 μL of PBS1X, the MNPs@anti-CD63 were washed with PBS1X three times via magnetic separation.

Biological samples of exosomes obtained from the Centre for Bionano Interaction Group (University College Dublin) were collected from the cell supernatant of the A549 cell line, where the protein concentration was estimated to be approximately 435.0 μg mL^−1^ by the MicroBCA assay. Then, 40.0 μL of the exosome sample was incubated with the above MNP@anti-CD63 solution for 2 h at 37 °C and 550 rpm. After magnetic washing, the MNPs@antiCD63@exosomes were lysed in RIPA buffer and subjected to DTT (1,4-dithiothreitol) reduction before SDS‒PAGE.

### Colorimetric titration by UV–visible spectra

To quantify the concentration of MNPs, 10.0 μL of MNPs solution was mixed with 20.0 µL of water and 90.0 µL of hydrochloric acid (37%), dissolving the nanoparticles in the form of ferric ion. Then, 2.9 mL of water, 60 µL of sodium thiocyanate solution (2 M), and 60.0 µL of the above sample were mixed under UV absorption at 461 nm, where a calibration curve of ferric ion was also recorded to estimate the MNP concentration.

To quantify the amine density of MNP@NH_2_, 200.0 µL of MNP was mixed with 250.0 µL of ninhydrin solution (20 mM) and acetic acid buffer (pH = 5.4) and incubated at 75 °C for 20 min. According to the UV absorption at 571 nm, the relevant intensity of the UV absorbance can be used to estimate the amount of amine groups, with a calibration curve of glycine.

### Characterization and equipments

MNPs@NH_2_ were diluted in ethanol to a concentration of 0.1 mg/mL, and 5.0 μL of MNPs was added to a 200-mesh copper transmission electron microscopy (TEM) grid with a carbon/formvar film (Ted Pella, Inc., Redding, CA, USA; Prod # 01754-F) for drying. Dynamic light scattering (DLS) in DI water or in PBS1X and zeta potential measurements in 10 mM HEPES buffer were performed using a Malvern Zetasizer Nano ZS instrument (Malvern Panalytical Ltd., UK). Differential centrifugal sedimentation (DCS) analysis of the MNPs was performed using a CPS Disk Centrifuge DC2400. The measurements were carried out using an 8 ~ 24% sucrose density gradient in DI water, with the disc speed set to 18,000 rpm while monitoring the 1 ~ 500 nm range. An EmStat3 USB-powered potentiostat was used for electrochemical characterization of the redox signal by square wave volumetry (SWV) and cyclic voltammetry (CV) with parameters such as a voltage ranging from 0 ~ 0.7 V, a 100 mV/s scanning rate, and 40 Hz. Protein characterization was performed via SDS‒PAGE (sodium dodecyl sulfate‒polyacrylamide gel electrophoresis) from Bio‒Rad with 8% gel preparation for protein electrophoresis with or without 10.0 mM DTT lysis.

## Results and discussion

### Amination and PEGylation of MNPs

**Fig. 2:**
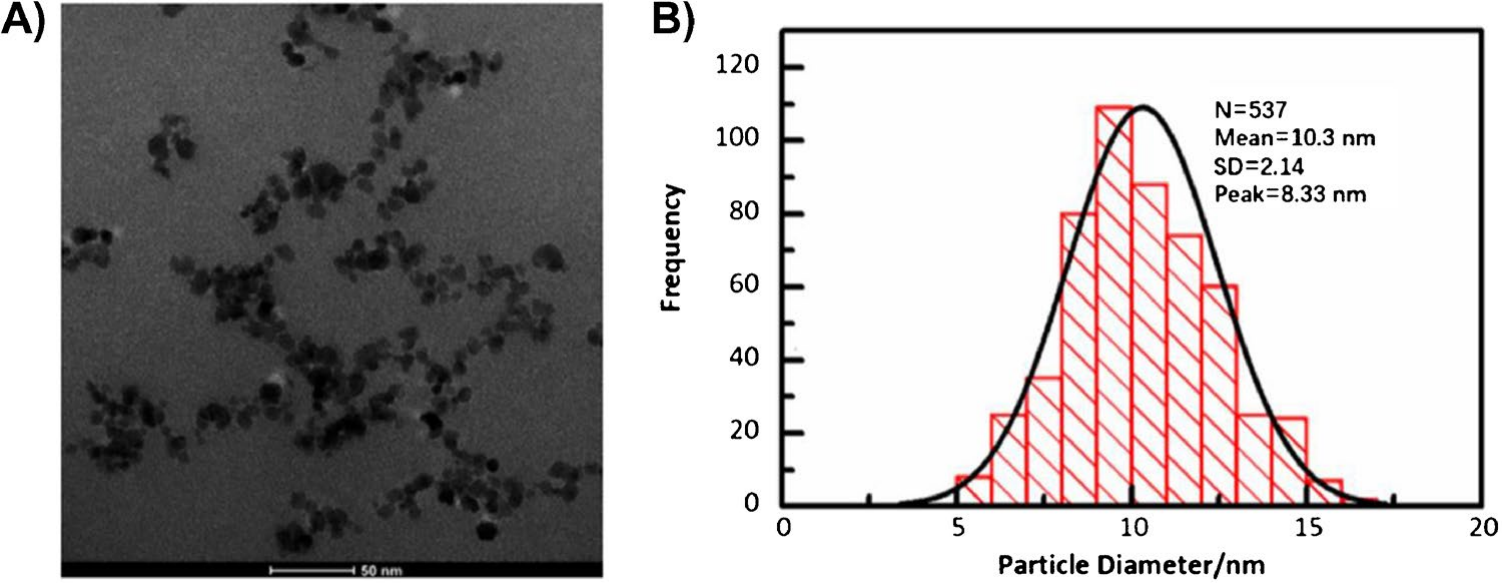
TEM characterization of MNPs@NH_2_. **A** TEM image. **B** TEM size distribution

**Fig. 3:**
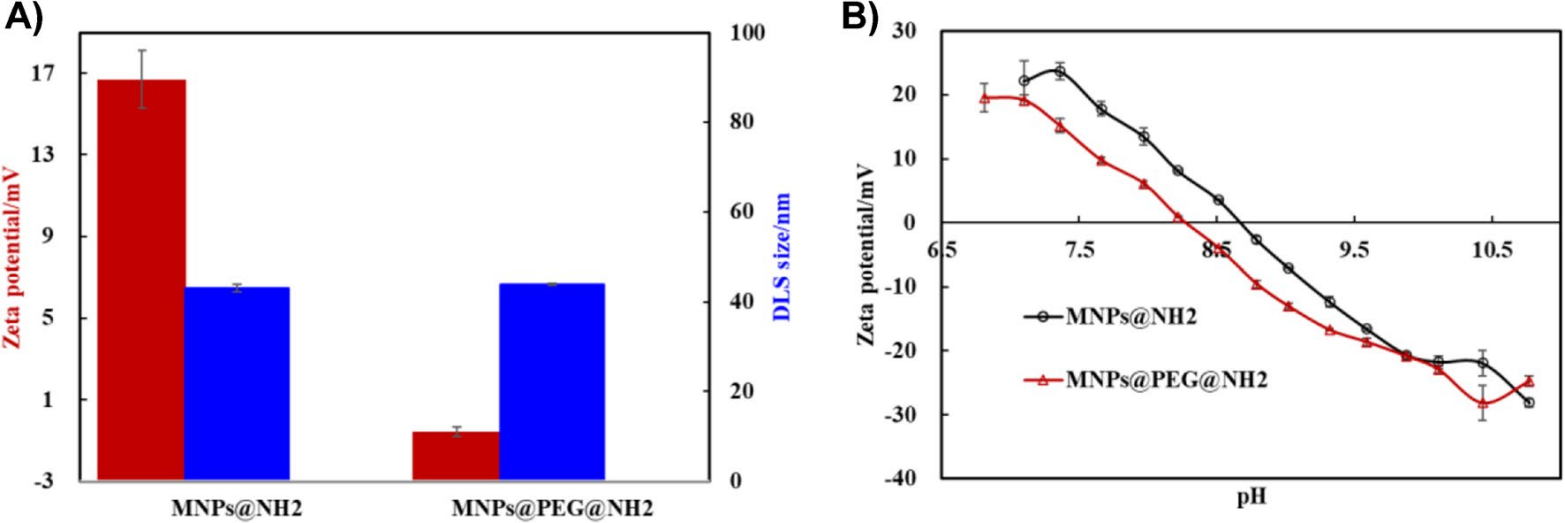
Zeta potential and DLS characterization of MNPs@NH_2_ and MNPs@PEG@NH_2_. **A** Zeta potential in 10.0 mM HEPES and DLS in DI water. **B** Zeta potential-pH titration of MNPs dispersed in a pH adjusted by using 10.0 mM nitric acid and 10.0 mM ammonium hydroxide

By co-precipitation method, iron(III) oxide dots (MNPs) were synthesized as small MNPs for this work [25–28]. The TEM image in Fig. 2A revealed the nano-size of MNP@NH_2_, while their size was estimated to be approximately 10.3 nm according to the histograms in Fig. 2B as measured by ImageJ software [29]. First, for the amination of APTMS, the surface properties were characterized by zeta potential measurements and DLS, as shown in Fig. 3A; the zeta potential of MNP@NH_2_ was measured to be 16.7 ± 1.42 mV in 10.0 mM HEPES, and its DLS was approximately 43.13 ± 0.85 nm in DI water. The amine density of MNP@NH_2_ was also determined to be approximately 3.34 ± 0.1 per nm^2^ by ninhydrin testing [30]. Additionally, after PEGylation, MNPs@PEG@NH_2_ presented a lower zeta potential (0.56 ± 0.23 mV) than MNP@NH_2_, revealing that the grafted PEGs at MNPs can increase the surface charge density needed to stabilize the MNPs. Although MNP@PEG@NH_2_ had a DLS size of 43.95 ± 0.22 nm, similar to that of MNPs@NH_2_, it prevented the aggregation of MNPs once dispersed in the high-ionic-strength buffer of PBS1X. In Fig. S1, the DLS size of MNPs@PEG@NH_2_ was approximately 60.79 ± 0.06 nm once dispersed in PBS1X. This finding confirmed the stability of the MNP dispersion owing to PEGylation at the MP surface. Additionally, zeta potential-pH titration curves of MNPs@NH_2_ and MNPs@PEG@NH_2_ were also recorded in the pH range of 6.5 to 11. As shown in Fig. 3B, the isoelectronic point of MNP@NH_2_ was approximately 8.65, but the isoelectronic point of MNP@PEG@NH_2_ was reduced to 8.22 because of PEGylation. We demonstrated that the presence of PEGs shifted the isoelectronic point of MNPs to maintain the high stability of MNP dispersions, particularly in high-ionic-strength buffers.

### BS3 cross-linking of IgG onto MNPs@PEG@NH_2_

**Fig. 4:**
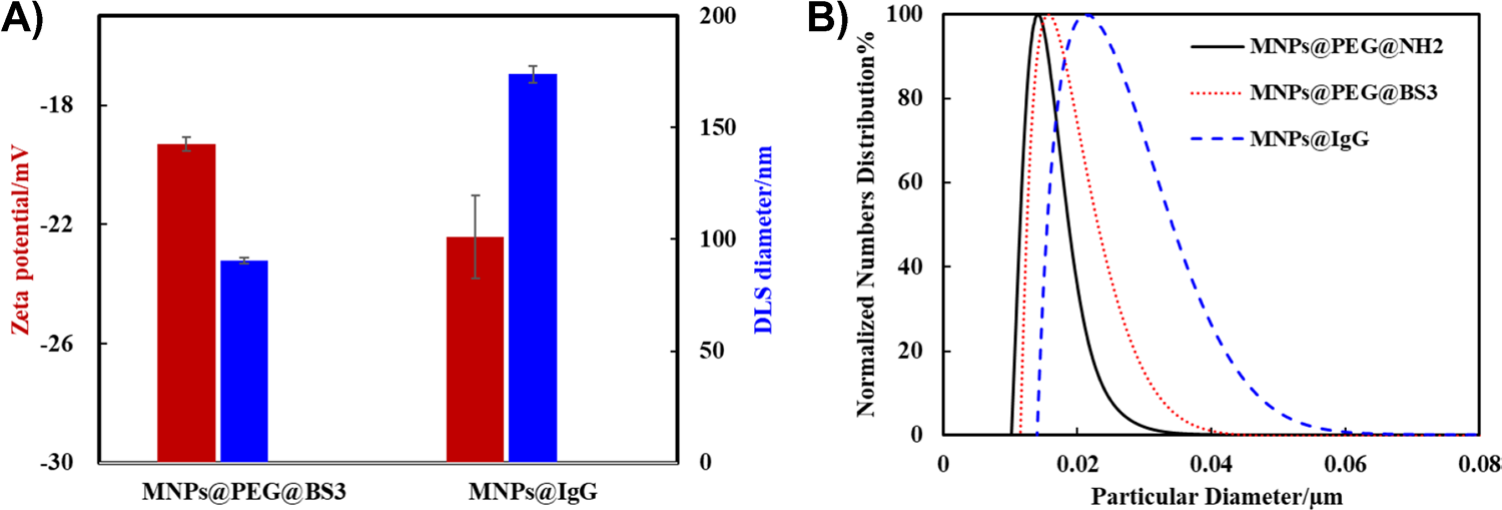
**A** Zeta potential (in 10 mM HEPES) and DLS size (in PBS1X) of MNP@PEG@BS3 and MNP@IgG.** B** DCS size distribution of MNP@PEG@NH_2_ with BS3 activation and IgG conjugation

The second step involved conjugating IgG onto the MNPs via the cross-linker of BS3 since BS3 contains two NHS-ester groups that can cross-link both IgG and MNPs@PEG@NH_2_. Compared to the zeta potential of MNPs@PEG@NH_2_ (0.56 ± 0.23 mV in Fig. 3A), it was notable that the zeta potential and DLS size of MNP@PEG@BS3 from Fig. 4A were determined to be − 19.2 ± 1.39 mV and 90.45 ± 1.29 nm, respectively, where the reduced zeta potential value was caused by the BS3 activating with amino groups located at the MNP@PEG@NH_2_ surface. However, the slight increase in the DLS size from MNPs@PEG@NH_2_ (43.95 nm) to MNPs@PEG@BS3 (90.45 nm) might be attributed to slight aggregation among the MNPs caused by BS3 crosslinking. Furthermore, the conjugation of IgG further decreased the zeta potential of the MNPs to − 22.43 ± 0.68 mV, while it increased their DLS size to 173.80 ± 3.83 nm. This result indicated that multiple crosslinking reactions occurred between the IgG and MNPs caused by the homobifunctional cross-linker of BS3. Interestingly, such multiple cross-linking phenomena did not cause high aggregation of the MNPs but seemed to be stabilized within a certain range of DLS sizes (< 200 nm).

In particular, the DCS diameter of the MNPs was also measured to confirm the aggregation level of the MNPs after BS3 activation and IgG conjugation. According to the distribution of DCS sizes in Fig. 4B, the peak size for MNP@PEG@NH_2_ was estimated to be 14.05 nm, which was attributed to monodispersed MNPs. After BS3 activation, the DCS peak of MNP@PEG@BS3 was shifted to 15.68 nm and had a wider particle distribution than that of MNP@PEG@NH_2_, indicating slight aggregation. However, after IgG conjugation, the DCS peak of the MNPs was located at 21.45 nm, corresponding to a wider size distribution. This demonstrated that BS3 crosslinking resulted in the formation of aggregated MNP@IgG, which were the complexes of IgG and MNPs.

**Fig. 5:**
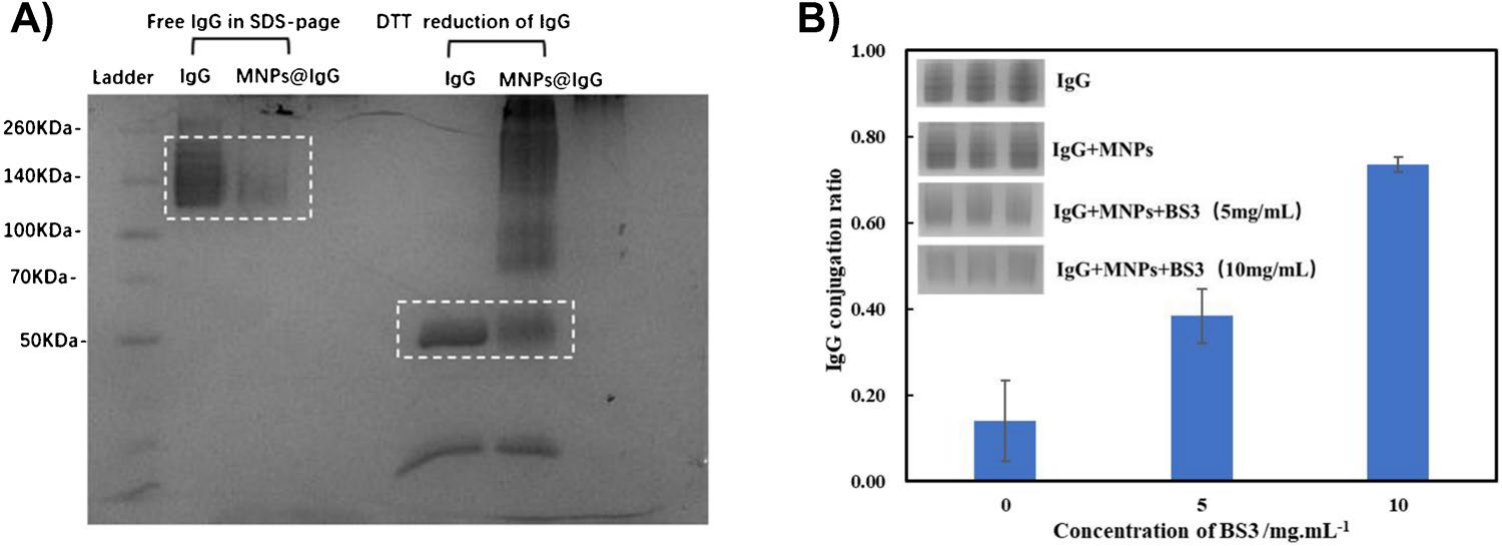
**A** Coomassie blue-stained SDS‒PAGE image of MNP@IgG with or without DTT reduction. **B** IgG conjugation efficiency onto MNP surfaces using different concentrations of the BS3 cross-linker (0, 5.0, and 10.0 mg/mL)

Additionally, to clarify the presence of these aggregated IgGs after conjugation, we further characterized the MNPs@IgG via SDS‒PAGE. Since free IgG can cross the gel and be stained with Coomassie blue, Fig. 5A showed that the gel bands corresponding to free IgG were observed at 140 kDa with a strong gel intensity consistent with Coomassie blue staining. When the MNPs@PEG@BS3 were incubated with free IgG, however, the prepared MNPs@IgG also presented a weak gel intensity at the same location as the gel bands. Because IgG conjugated onto the MNP surface cannot cross the gel for analysis, only a weak band associated with the small amount of free IgG was observed at 140 kDa. And the majority of the IgG was conjugated onto the MNPs, resulting in a reduced gel intensity at 140 kDa. To analyse the conjugated IgG by SDS‒PAGE, the reducing reagent of DTT was used to reduce the conjugated IgG to a heavy chain of 50 kDa and a light chain of 25 kDa, so that these conjugated IgG can be released from the MNP surface for SDS‒PAGE imaging [31]. Indeed, these free IgGs were successfully reduced to the heavy chain and light chains, as shown by the corresponding gel band locations in the SDS‒PAGE image in Fig. 5A. However, the MNP@IgG sample exhibited a large amount of gel bands between 260 and 70 kDa after DTT reduction, in addition to the typical gel bands at 50 kDa and 25 kDa. According to the DLS measurements (Fig. 4A) of the MNP@IgG complex, we realized that BS3-mediated crosslinking occurred not only among the MNPs and IgG but also among these IgG molecules. Due to the non-reducing cross-linker BS3, the crosslinking among these IgGs cannot be completely reduced by DTT, which resulted in the formation of long trailing gel bands, as displayed in Fig. 5A.

Furthermore, in terms of the optimized BS3 concentration, Fig. 5B showed the evaluation of the binding efficiency of the IgG and MNPs when three concentrations of BS3 were used for preparation, such as 0, 5.0, and 10.0 mg/mL. The non-specific adsorption between MNPs and IgG was estimated to be approximately 0.14 ± 0.09. However, with 5.0 mg/mL BS3, the conjugation ratio of IgG increased to 0.38 ± 0.06, but at a concentration of 10.0 mg/mL BS3, the conjugation ratio further improved to 0.73 ± 0.02. A further high concentration of BS3 used here can cause visible aggregation of MNPs@IgG. To validate the possibility of the aggregation issue mediated by the increasing amount of BS3 in co-crosslinking method, we further evaluated the DLS and DCS size of MNPs@IgG at 5.0 mg/mL of BS3. In Figure S2, the DLS size of MNPs@IgG was increased with the increase of BS3 amount. Meanwhile, their DCS size distribution exhibited an increasing aggregation level at the size scale between 50 and 70 nm. Thus, we believed that the optimal concentration of BS3 used in this work should be 10.0 mg/mL.

### Co-crosslinking of the redox probe and IgG onto MNPs

**Fig. 6:**
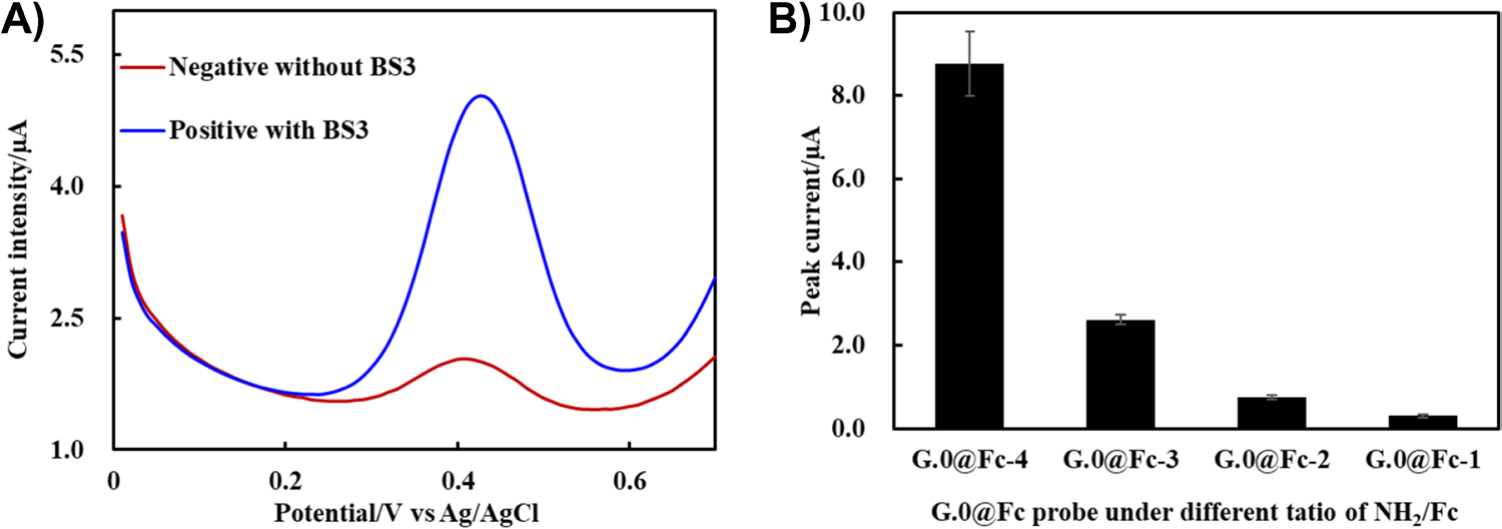
**A** Redox labelling efficiency of MNPs@Redox@IgG (200 μL, 1.0 mg/mL) with or without BS3 co-cross-linking. **B** Redox intensity of MNPs@Redox@IgG generated by using different G.0@Fc probes (at different ratios of amine to Fc from 1 to 4)

Fc, as a redox probe model, was firstly conjugated onto the support of the PAMAM G.0 surface to form amine based on the redox probe G.0@Fc (Figure S3A). As PAMAM G.0 contains four amine groups, it enabled the synthesis of the redox probe G.0@Fc with 1 to 4 of Fc conjugates, such as G.0@Fc-1, G.0@Fc-2, G.0@Fc-3, and G.0@Fc-4, depending on the molecular ratio of Fc/G.0. Figure S3B showed that the oxidation peak of Fc and G.0@Fc-2 shifted from 0.57 to 0.45 V, indicating that these Fc moieties were covalently conjugated onto the PAMAM surface [32]. By using the BS3 cross-linker, both G.0@Fc-2 and IgG were co-conjugated onto the MNP surface to achieve dual functionalization of the MNPs. As a negative control in Fig. 6A, the redox intensity of the MNPs without BS3 was reported to be approximately 0.56 ± 0.02 μA, but it was measured to be 3.28 ± 0.15 μA after BS3 co-crosslinking. This result indicated that the redox probe G.0@Fc-2 was co-crosslinked onto the MNP surface. Moreover, the presence of IgG was also confirmed by SDS‒PAGE, as shown in Figure S4A, where the reduced IgG presented two gel bands at 50 kDa and 25 kDa, similar with the previous Fig. 5A. Therefore, we believed that our strategy of co-crosslinking strategy can be used to construct dual-functionalized MNPs with biological and redox properties.

Additionally, to optimize the redox intensity of dual-functionalized MNPs, all of the redox probes G.0@Fc with different Fc ratios were also selected for preparation of the dual-functionalized MNPs. According to the SWV curves, the oxidation peaks of the MNPs were all located at 0.44 V (Figure S5). The redox intensity of these dual-functionalized MNPs@Redox@IgG increased from that of G.0@Fc-1 to that of G.0@Fc-4, as presented in Fig. 6B, where a high amount of Fc conjugates can offer a high redox intensity to the MNPs. In particular, as G.0@Fc-4 enabled the maximization of the redox intensity of MNPs, G.0@Fc-4 probes might also mainly adsorb onto IgG surfaces rather than dominated by the co-crosslinking process.

### DTSSP cross-linking of IgG onto MNPs

Although SDS‒PAGE can be used for bio-testing by investigating the bio-nano interactions between exosomes and antibody-based MNPs, the gel band trailing observed at 260 to 70 kDa in Fig. 5A might be a challenge for using SDS‒PAGE in further studies. To address this issue, we utilized DTSSP instead of BS3 for the co-crosslinking of IgG since the cleavable DTSSP can be completely reduced by DTT. This approach can eliminate these unwanted gel bands of SDS‒PAGE observed in IgG crosslinking and can avoid the potential interference in gel bands analysis.

**Fig. 7:**
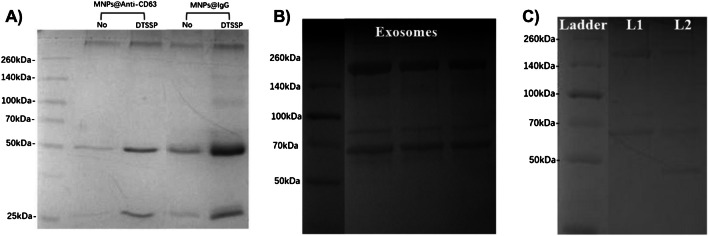
**A** Coomassie blue-stained SDS‒PAGE of MNPs@anti-CD63 and MNPs@IgG with DTT reduction. **B** Coomassie blue-stained SDS‒PAGE of exosomes after RIPA buffer lysis and DTT reduction. **C** Coomassie blue-stained SDS‒PAGE of exosome samples and MNP@anti-CD63-extracted exosomes (L1: 10.0 μL of exosome sample, L2: exosome extracted by MNPs@anti-CD63)

In Fig. 7A, MNP@IgG only presented bands corresponding to the heavy chain and light chain at 50 kDa and 25 kDa, respectively, and no additional gel bands were observed in the SDS‒PAGE gel, indicating that the DTSSP cross-linker was completely reduced by DTT, preventing the gel bands from the trailing among 70 ~ 260 kDa. MNPs@PEG@NH_2_ were mixed with IgG directly as a negative control, while an amount of DTSSP was added to the mixture of MNPs@PEG@NH_2_ and IgG for cross-linking as a positive control. As presented in Fig. 7A, DTSSP cross-linker-induced IgG conjugation resulted in more intense gel bands than that of non-specific adsorption (Figure S6). Furthermore, the DLS sizes (Figure S7A) of MNP@IgG and MNP@Redox@IgG were estimated to be 155.2 ± 2.78 nm and 178.6 ± 4.1 nm, respectively, which were similar to those of BS3 used in the co-crosslinking strategy. And as characterized by SWV, the redox signal of MNPs@Redox@IgG was shown in Figure S7B, where the oxidation peak of MNPs was located at 0.44 V and the signal intensity was 10.15 ± 0.54 μA.

### Bio-application of MNPs@anti-CD63

To access their bio-nano interactions, anti-CD63 antibody was conjugated onto the MNP surface by using the DTSSP cross-linker, according to our co-crosslinking strategy. As validated by SDS‒PAGE, as shown in Fig. 7A, there were clear gel bands at 50 kDa for the heavy chain, indicating that the anti-CD63 antibody was conjugated onto the MNP surface. In the bio-testing, exosome samples from the A549 cell supernatant were collected by the ultra-centrifugation method. As characterized by SDS‒PAGE, the exosome samples were first lysed in RIPA buffer and then reduced by DTT, as shown in Fig. 7B. Two gel bands corresponding to the exosomes were observed at 200 kDa and 70 kDa, revealing that the exosomes contained the different kinds of the proteins. To clarify the targeted biomarker of CD63 from exosomes, western blotting was utilized to confirm the location of the gel associated with the CD63 biomarker, as shown in Figure S8. This result indicated that only one band was clearly located at 70 kDa, which may be related to the presence of the CD63 biomarker [33, 34]. When the MNPs@anti-CD63 were used for exosome capture, the extracted exosomes were also subjected to SDS‒PAGE. SDS‒PAGE revealed one band corresponding to the exosome biomarker of CD63 at 70 kDa and another band corresponding to the heavy chains of CD63 antibody at 50 kDa (Fig. 7C), demonstrating that these anti-CD63 antibodies enabled the immunologically affinity of these MNPs for exosome capture after crosslinking onto the MNP surface.

## Conclusions

Dual-functionalized MNPs featured with both of redox amplification and bio-recognition, can be an aide of the diagnostic assay or electrochemical biosensing, because they will be capable of specifically capturing targets, magnetically separating targets and electrochemically sensing for bio-application. In particular, it will be compatible in an integrated diagnostic device, like point-of-care assay with a “sample-in-answer-out” manner. To achieve this goal, we developed a co-crosslinking strategy as a robust surface functionalization method for construction of dual-functionalized MNPs, from the synthesis and surface functionalization of MNPs to the co-crosslinking of redox and biological probes. Clearly, the co-crosslinking methods will be a bridge from the nanomaterials to their broad bio-application. On the one hand, the synthesized MNPs with a naked surface were aminated with APTMS to attach to amine groups and then further PEGylated to improve their colloid stability in PBS1X. On the other hand, the biological probe IgG and the redox probe Fc were co-crosslinked onto the MNP surface by the BS3 cross-linker, as successfully confirmed by SDS‒PAGE and SWV. The IgG binding efficiency was estimated to be approximately 73%, mediated by BS3, and the redox intensity of the MNPs was electrochemically measured to be approximately 10.15 ± 0.54 μA. In bio-applications, the anti-CD63 used to construct dual-functionalized MNPs exhibited bio-affinity for capturing exosomes. Additionally, as a common method, the co-crosslinking strategy proposed here might potentially lead to new ways to construct dual-functionalized MNPs for their use in broad biosensing system, such as electrochemical, fluorescent, and plasmonic-based biosensing system.

### Supplementary Information

Below is the link to the electronic supplementary material.

Supplementary file1 (RTF 21852 KB)

## Data Availability

The data are available from the corresponding author upon reasonable request.
