# Supplementary material for: Co-crosslinking strategy for dual functionalization of small magnetic nanoparticles with redox probes and biological probes
Source: Mikrochim Acta. 2024 Jul 5;191(8):448. doi: 10.1007/s00604-024-06517-8 (PMC11229446; doi:10.1007/s00604-024-06517-8)
Supplement: Supplementary file 1 — Supplementary file1 (RTF 21852 KB) [file 604_2024_6517_MOESM1_ESM.rtf]

Supplementary materials
Co-crosslinking strategy for dual functionalization of small magnetic nanoparticles with redox probes and biological probes

Ye Chena and Feixiong Chen*b,c
*Corresponding author: feixiong.chen@oulu.fi and chenfeixiong1988@gmail.com

a. Huangyan District Center for Disease Control and Prevention, Taizhou, Zhejiang, China
b. Centre for BioNano Interactions, School of Chemistry and Chemical Biology, University College Dublin, Belfield, Dublin 4, Ireland
c. Faculty of Biochemistry and Molecular Medicine, University of Oulu, 90014 Oulu, Finland


Figure S1. DLS size of MNPs@PEG@NH2 in PBS1X


Figure.S2 Aggregation level of MNPs@IgG prepared at different BS3 (using concentration of 5.0 mg/mL BS3) volumes. A) DLS size (in PBS1X) of MNPs@IgG. B) DCS size distribution of MNPs@IgG.


Figure S3. A) Conjugation of Fc onto the PAMAM G.0 surface at different ratios. B) Cyclic voltammetry of Fc and G.0@Fc-2


Figure S4. A) SDS‒PAGE of MNP@Redox@IgG with or without DTT reduction. B) IgG conjugation efficiency onto the MNP surface by using different concentrations of the BS3 cross-linker.


Figure S5. SWV curves of MNPs@Redox@IgG obtained by using the redox probes of G.0@Fc-1, G.0@Fc-2, G.0@Fc-3, and G.0@Fc-4.


Figure S6. SDS‒PAGE gel intensities of MNP@anti-CD63 and MNP@IgG with and without DTSSP crosslinking are shown in Figure 7A.


Figure S7. A) DLS sizes of MNP@IgG and MNP@Redox@IgG obtained by using the DTSSP cross-linker. B) SWV curves of MNP@Redox@IgG obtained by using G.0@Fc-4 under DTSSP Co-crosslinking.


Figure S8. Characterization of exosomes by Western blot using CD63 antibody and goat anti-mouse IgG HRP.
